# Implicit transitive inference and the human hippocampus: does intravenous midazolam function as a reversible hippocampal lesion?

**DOI:** 10.1186/1744-9081-3-51

**Published:** 2007-09-24

**Authors:** Anthony J Greene

**Affiliations:** 1 Department of Psychology, University of Wisconsin, Milwaukee, Milwaukee, WI, USA

## Abstract

Recent advances have led to an understanding that the hippocampus is involved more broadly than explicit or declarative memory alone. Tasks which involve the acquisition of complex associations involve the hippocampus whether the learning is explicit or implicit. One hippocampal-dependent implicit task is transitive inference (TI). Recently it was suggested that implicit transitive inference does not depend upon the hippocampus (Frank, M. J., O'Reilly, R. C., & Curran, T. 2006. When memory fails, intuition reigns: midazolam enhances implicit inference in humans. *Psychological Science, 17*, 700–707). The authors demonstrated that intravenous midazolam, which is thought to inactivate the hippocampus, may enhance TI performance. Three critical assumptions are required but not met: 1) that deactivations of other regions could not account for the effect 2) that intravenous midazolam does indeed deactivate the hippocampus and 3) that midazolam influences explicit but not implicit memory. Each of these assumptions is seriously flawed. Consequently, the suggestion that implicit TI does not depend upon the hippocampus is unfounded.

## Introduction

The capacity to learn complex stimulus relations and express them flexibly is thought to require the hippocampus [1]. While the hippocampus has been thought to be necessary for explicit but not implicit forms of learning and memory [2], more recent studies demonstrate hippocampal involvement in implicit tasks which require complex associations [3-8]. One task which involves both the acquisition of complex relations, flexible expression and which may be learned implicitly is the transitive inference task [8-13]. At study, participants learn complex relations among stimuli (e.g., A>B, B>C, C>D, D>E) and at test, they show flexible expression when an untrained discrimination (B?D) is correctly solved (B>D). Functional imaging studies confirm the role of the hippocampus in both implicit and explicit versions of TI [8,14-16] and, amnesic patients with damage to the hippocampus and surrounding tissue cannot perform the TI task [17].

A recent study demonstrated that the administration of intravenous midazolam improved TI performance in human participants [18] which was argued as evidence that TI is performed more easily when the hippocampus does not contribute explicit strategies. The authors argue that midazolam deactivates the hippocampus because benzodiazepines act on GABA-A receptors [19], which are densely concentrated in the hippocampus [20], and because benzodiazepines – in particular midazolam – temporarily impair explicit memory formation [21]. The assertion that intravenous midazolam improves TI performance by deactivating the hippocampus depends entirely upon three premises: 1) that other areas critical to TI are not affected, so that only hippocampal deactivation can explain the effect; 2) that midazolam deactivates the hippocampus to the point that it no longer functions in a mnemonic capacity; and 3) That midazolam influences explicit but not implicit memory. There are serious problems with all three premises, each of which jeopardize the claim that TI is performed more easily without the hippocampus [18].

## Discussion

In studies where midazolam has been shown to have selective effects on the hippocampus, non-human animals receive midazolam as a direct injection into the hippocampal tissue [22]. The principle action of midazolam is as a central nervous-system depressant, which is frequently used medicinally as a sedative or as an anti-convulsant. While midazolam may be useful as a reversible lesion when injected directly into the hippocampus, it is not at all clear that the effects are comparable when it is injected intravenously. One might expect the action of midazolam to be much less circumscribed when it is distributed globally. Indeed, midazolam has been shown to decrease regional cerebral blood flow (rCBF) to numerous cortical areas other than the hippocampus or surrounding cortices [23]. Thus, it is entirely possible that the effects of midazolam on the TI task are attributable to deactivations of areas other than the hippocampus.

Of the regions shown in human imaging studies to be deactivated by normal concentrations of intravenous midazolam, the hippocampus is not among them. Two studies attempting to discover an effect of midazolam on rCBF in the hippocampus found no such effects [23,24]. Clearly, failure to detect an effect of midazolam on the rCBF of the hippocampus does not imply the effects do not exist. However, given a compromised hippocampus can still mediate hippocampal-dependent tasks [25] the burden of proof must be upon those who claim that intravenous midazolam anesthetizes the hippocampus to the extent that it does not function effectively in a mnemonic capacity. Moreover, a third study varied the concentration of intravenous midazolam and showed a global anesthetizing effect at very high dosages to which the hippocampus and parahippocampal regions were particularly resistant compared to other regions of cortex [26]. It would, therefore, strain credibility to argue that the effects of intravenous midazolam on the hippocampus account for its amnesic effects but simply do not reach significance when measured by rCBF.

Furthermore, were it the case that midazolam selectively deactivates the hippocampus, one would expect the effects of midazolam on TI [18] to yield performance levels comparable to those observed in amnesic patients, but quite the contrary, amnesics are profoundly impaired on the TI task [17]. It is unclear from amnesic performance whether their inability to perform the inference portion of the task was predicated by their inability to acquire the premise pairs [17]. That is, amnesics do not reliably learn that B>C and C>D and that alone could account for their inability to make the correct inference B>D. While the amnesic data do not therefore allow strong conclusions about performance on just the inference portion of the task, they do tend to disconfirm the hypothesis that "midazolam should improve TI test performance by preventing participants from memorizing the stimulus pairs and encouraging greater implicit learning of reinforcement value" [[18] p. 701]. Furthermore, performance differences between hippocampal amnesia and midazolam-induced amnesia provide further evidence that the effects of intravenous midazolam are unlikely to constitute a hippocampal deactivation.

Given the widespread effects of intravenous midazolam in regions of cortex other than the hippocampus, it is natural to question whether the effects of midazolam are limited to explicit memory. Several studies show that midazolam influences not just explicit, but also implicit memory performance [27,28]. While implicit memory is not as profoundly affected as explicit memory – indeed, explicit performance drops to chance levels – it is nevertheless incorrect to assert that intravenous midazolam does not affect implicit performance.

## Conclusion

The existing data do not permit claims about selective effects if intravenous midazolam on the human hippocampus. Rather, the effects of intravenous midazolam involve numerous regions of cortex, including frontal and parietal regions. The assertion that intravenous midazolam selectively deactivates the hippocampus is at best premature and is more likely to be incorrect. Therefore, there is no adequate substantiation for the claim that TI is improved by hippocampal deactivation [18].

## Competing interests

The author(s) declare that they have no competing interests.
